# Supplementary figures and images for: Dissecting the respective roles of microbiota and host genetics in the susceptibility of Card9−/− mice to colitis
Source: Microbiome. 2024 Apr 23;12:76. doi: 10.1186/s40168-024-01798-w (PMC11036619; doi:10.1186/s40168-024-01798-w)

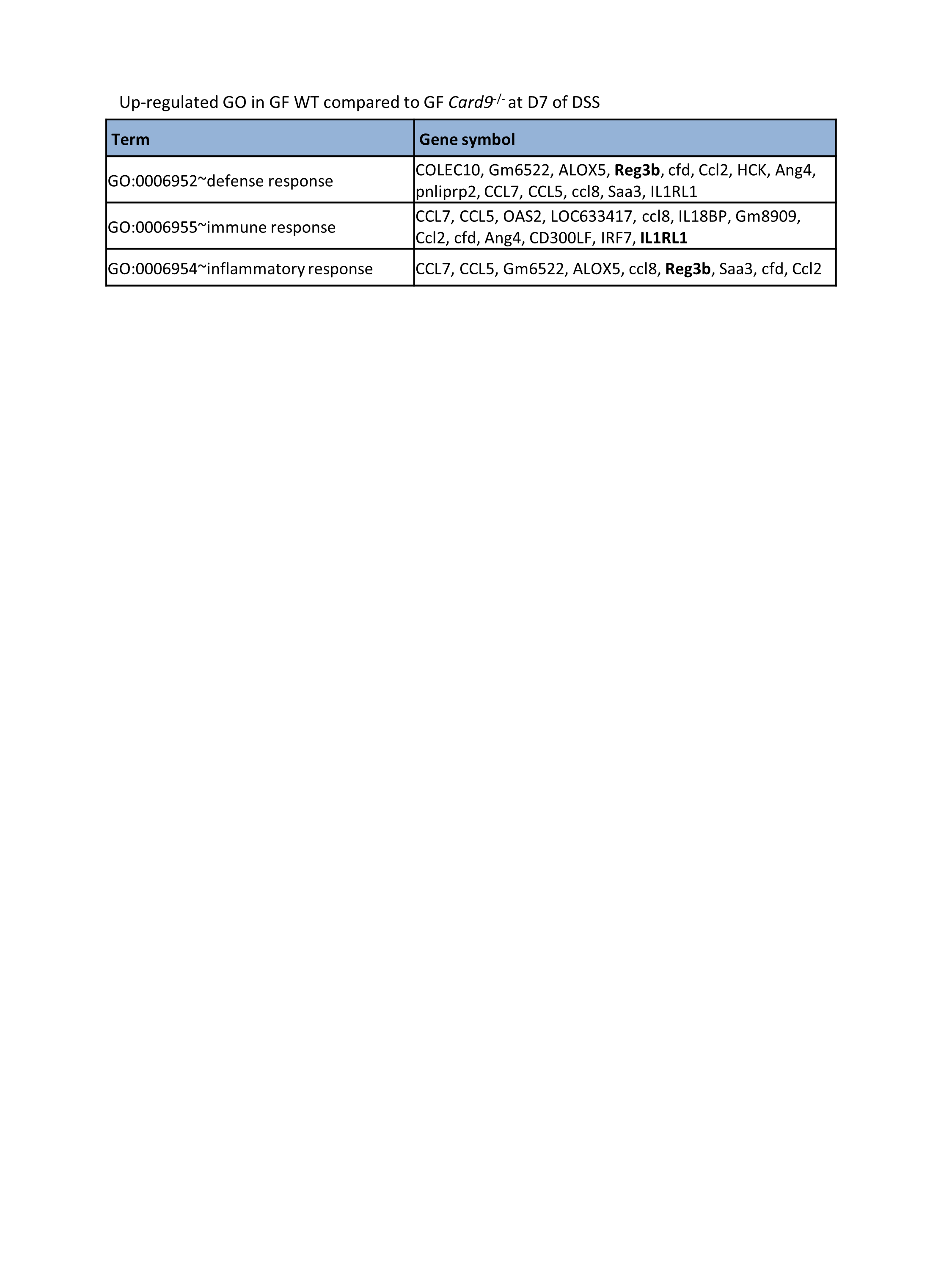

Supplement: Supplementary file 2 — Additional file 1: Supp Figure 1. Genes included in the Gene Ontology pathways downregulated in the colon of GF Card9-/- versus GF WT mice at day 7 of colitis (host defense (GO:0006952), immune response (GO:0006955) and inflammatory response (GO:0006954)), including Reg3β and Il1rl1. [file 40168_2024_1798_MOESM1_ESM.tif]

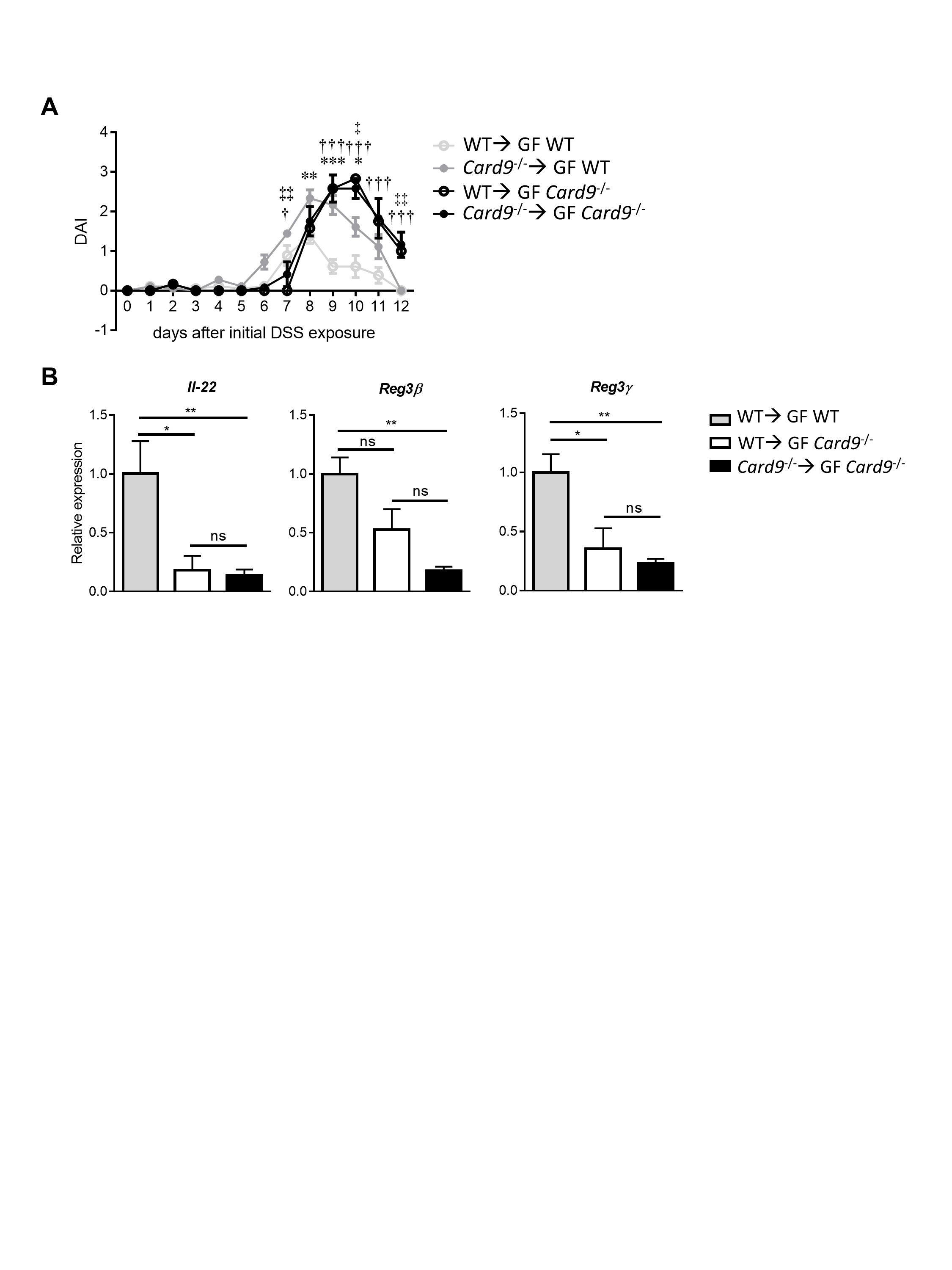

Supplement: Supplementary file 3 — Additional file 2: Supp Figure 2. (A) Disease activity index (DAI) of DSS-exposed GF Card9-/- or GF WT mice colonized with the microbiota of WT or Card9-/- mice. For statistical comparisons, † indicates WT ➔ GF WT versus WT ➔ GF Card9-/-, ‡ indicates Card9-/- ➔ GF WT versus Card9-/- ➔ GF Card9-/- and * indicates WT ➔ GF WT versus Card9-/- ➔ GF WT. (B) Il-22, Reg3β and Reg3γ expression by qRT-PCR in total colon tissue of WT ➔ GF WT, WT ➔ GF Card9-/- and Card9-/- ➔ GF Card9-/- mice at day 12, normalized to Gapdh. Data are mean ± SEM. *P<0.05, †P<0.05, ‡ P<0.05, **P<0.01, ‡‡P<0.01, ***P<0.001 and †††P<0.001 as determined by one way ANOVA and post hoc Tukey test (A) and Mann-Whitney test (B). [file 40168_2024_1798_MOESM2_ESM.tif]

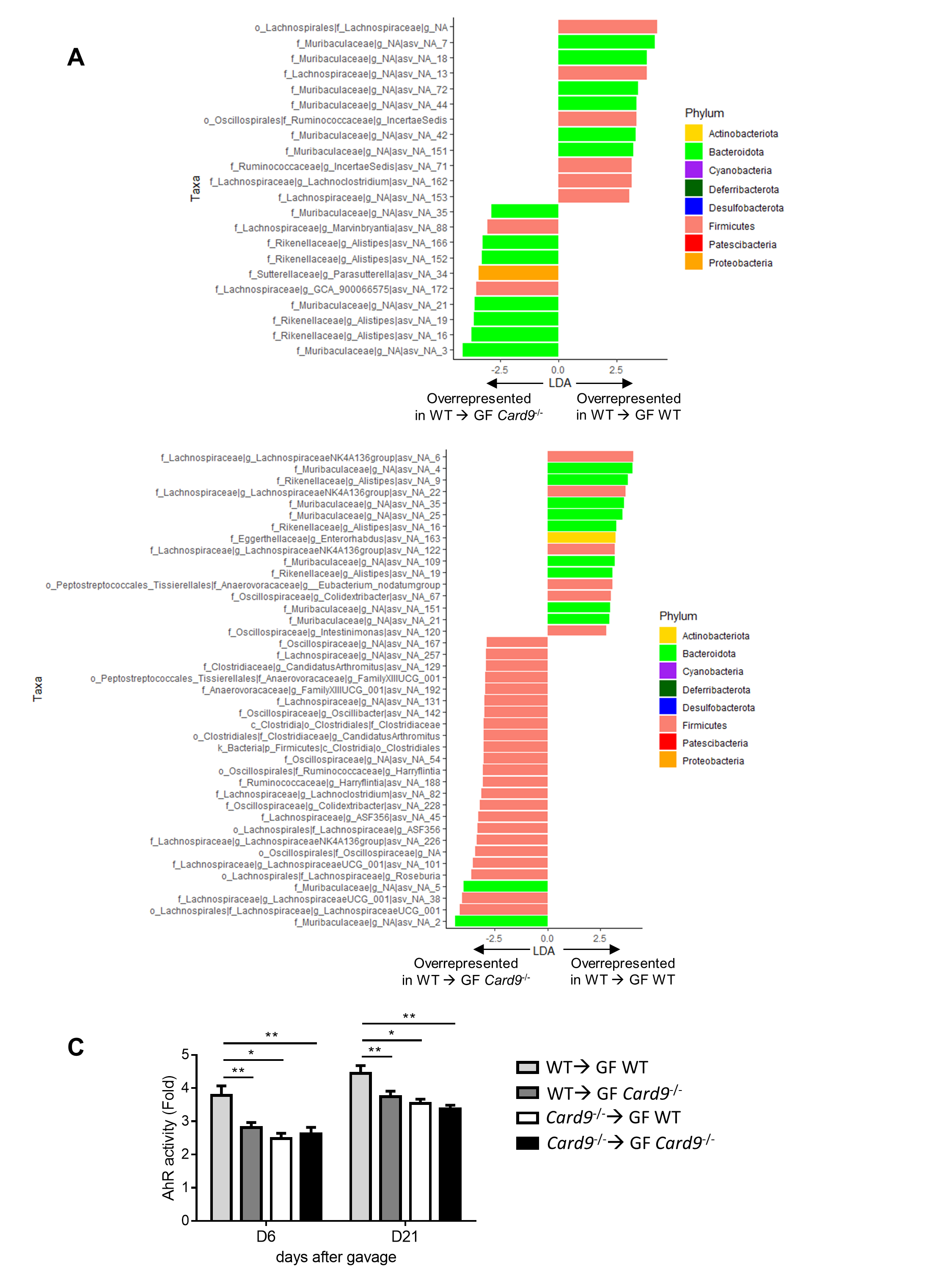

Supplement: Supplementary file 4 — Additional file 3: Supp Figure 3. LEFse analyses showing taxa (genus level) overrepresented (positive values, right) and underrepresented (negative values, left) in the microbiota of WT ➔ GF WT compared to WT ➔ GF Card9-/- mice at day 7 (A) and 21 (B). (C) AHR activity (shown as fold change) of feces of WT ➔ GF WT, Card9-/- ➔ GF WT, WT ➔ GF Card9-/- and Card9-/- ➔ GF Card9-/- mice at day 6 and 21. Data are mean ± SEM. *P<0.05 and **P<0.01, as determined by Mann-Whitney test. [file 40168_2024_1798_MOESM3_ESM.tif]

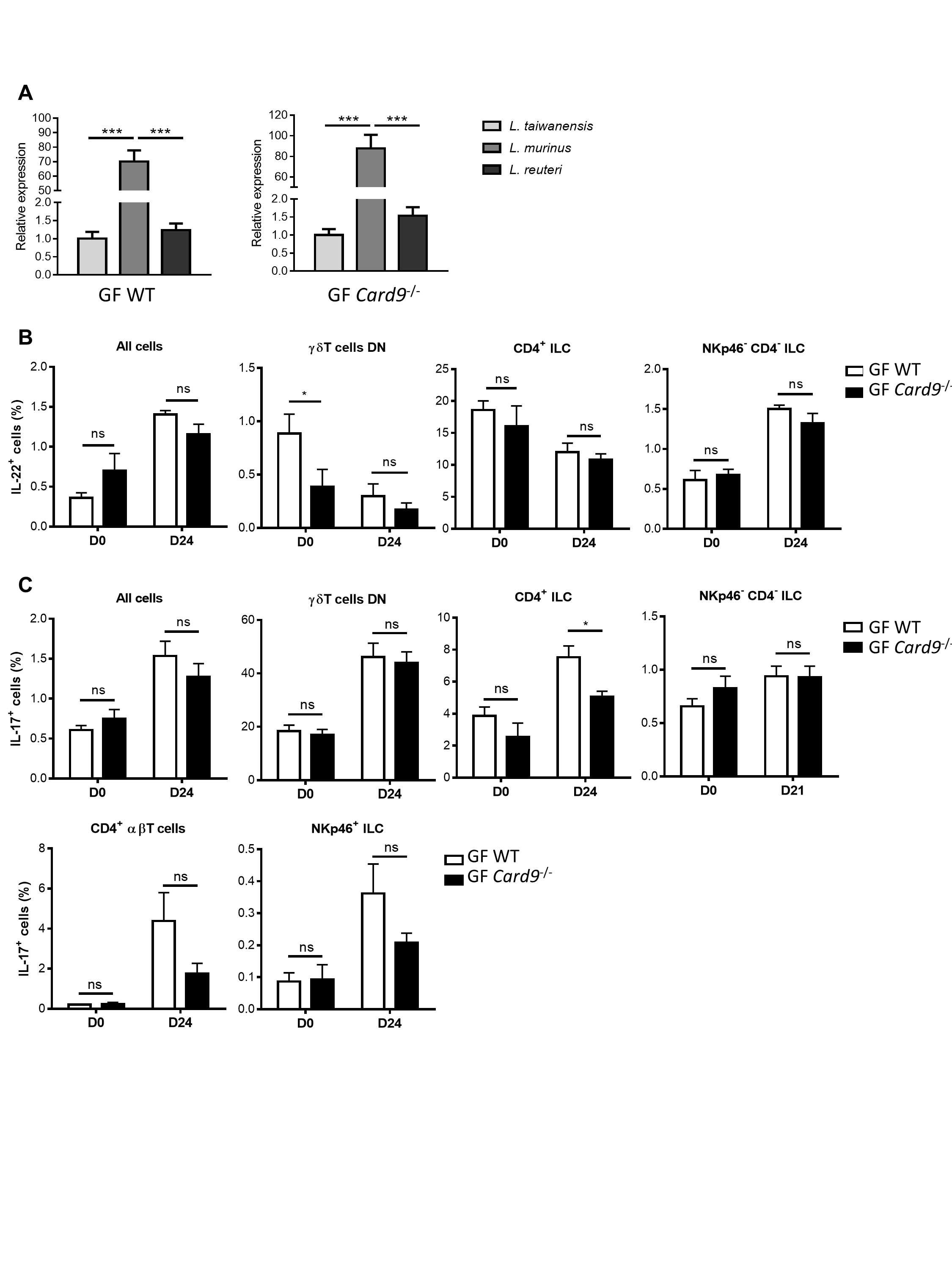

Supplement: Supplementary file 5 — Additional file 4: Supp. Figure 4. (A) Relative expression of each of the three gavaged Lactobacillus strains (L. murinus CNCM I-5020, L. reuteri CNCM I-5022 and L. taiwanensis CNCM I-5019) in feces of GF WT (left) and GF Card9-/- mice (right), assessed by qRT-PCR and normalized to "All lactobacilli" quantity at day 24. (B) Percentage of IL-22+ cells among all immune cells, γδT cells DN, CD4+ ILCs, and NKp46- CD4- ILCs in the colon lamina propria of GF WT and GF Card9-/- mice, at day 0 and 24 after gavage with the three Lactobacillus strains. (C) Percentage of IL-17+ cells among all cells, γδT cells DN, CD4+ ILCs, NKp46- CD4- ILCs, CD4+ αβT cells and NKp46+ ILCs in the colon lamina propria of GF WT and GF Card9-/- mice, at day 0 and 24 after gavage with the three Lactobacillus strains. Data points represent individual mice. Data are mean ± SEM. *P<0.05, **P<0.01, ***P<0.001, as determined by Mann-Whitney test. DN, double negative. [file 40168_2024_1798_MOESM4_ESM.tif]

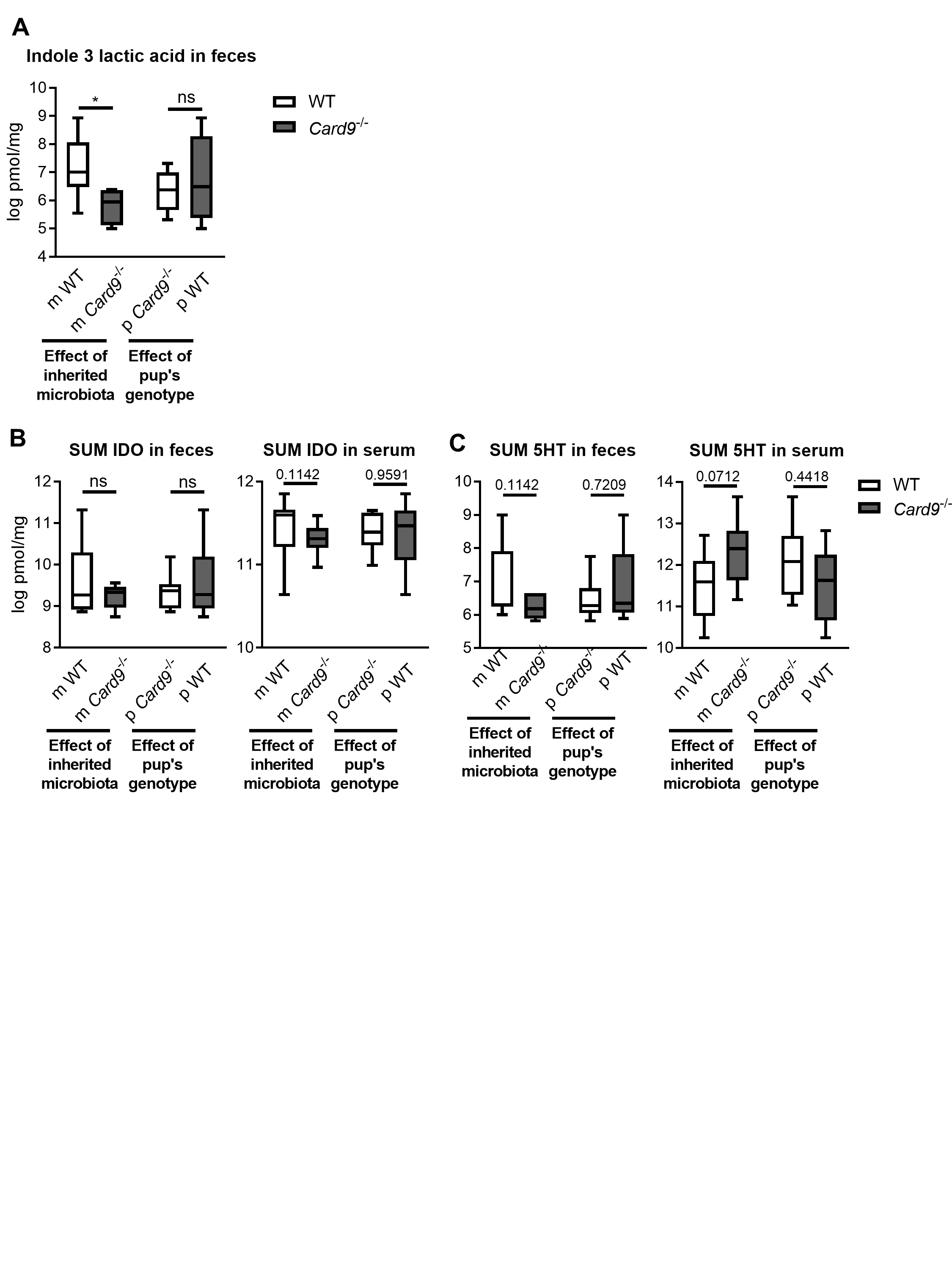

Supplement: Supplementary file 6 — Additional file 5: Supp Figure 5. (A) Indole 3 lactic acid concentration in feces of the pups separated according to the nursing mother genotype or the pups' genotype 5 weeks after weaning. Metabolites concentration from the IDO (B) and serotonin (5HT, C) pathways measured in feces or serum of the pups separated according to the nursing mother genotype or the pups' genotype, 5 weeks after weaning. Data points represent individual mice. *P<0.05, as determined by Mann-Whitney test. Trp, tryptophan. [file 40168_2024_1798_MOESM5_ESM.tif]
