# Supplementary material for: Dissecting the respective roles of microbiota and host genetics in the susceptibility of Card9−/− mice to colitis
Source: Microbiome. 2024 Apr 23;12:76. doi: 10.1186/s40168-024-01798-w (PMC11036619; doi:10.1186/s40168-024-01798-w)
Supplement: Supplementary file 7 — Additional file 6: Supplemental Table 1. Antibodies and nucleotides list. [file 40168_2024_1798_MOESM6_ESM.docx]

**Supplementary table 1**. Antibodies and oligonucleotides list

| **Antibodies** | **Source** |
| --- | --- |
| CD3 (145-2C11) | eBioscience |
| CD4 (L3T4) | BD Difco |
| CD8α (53-6.7) | Biolegend |
| CD11b (M1/70) | eBioscience |
| CD11c (N418) | eBioscience |
| F4/80 (BM8) | eBioscience |
| CD103 (M290) | BD Difco |
| MHC II (M5/114.15.2) | BD Difco |
| TCR-γδ (eBioGL3) | eBioscience |
| NKp46 (29A1.4) | eBioscience |
| IL-17A (TC11-18H10) | BD Difco |
| and IL-22 (IL-22JOP) | eBioscience |
|  |  |
| **Oligonucleotides** | **Sequence** |
| Gapdh (sense) | 5′-AACTTTGGCATTGTGGAAGG-3′ |
| Gapdh (antisense) | 5′-ACACATTGGGGGTAGGAACA-3’ |
| Il17a (sense) | 5′-TTTAACTCCCTTGGCGCAAAA-3′ |
| IL17a (antisense) | 5′-CTTTCCCTCCGCATTGACAC-3′; |
| Il22 (sense) | 5′-CATGCAGGAGGTGGTACCTT-3′ |
| Il22 (antisense) | 5′-CAGACGCAAGCATTTCTCAG-3′; |
| Reg3g (sense) | 5′-TTCCTGTCCTCCATGATCAAAA-3′ |
| Reg3g (antisense) | 5′-CATCCACCTCTGTTGGGTTCA-3 |
| Reg3b (sense) | 5’-ATGCTGCTCTCCTGCCTGATG-3′ |
| Reg3b (antisense) | 5′-CTAATGCGTGCGGAGGGTATATTC-3′ |
| *L. murinus* (sense) | 5’-GCAATGATGCGTAGCCGAAC-3’ |
| *L. murinus* (antisense) | 5’-GCACTTTCTTCTCTAACAACAGGG-3’ |
| *L. taiwanensis* (sense) | 5’-CAACGGATATAAGACAACACTCATGACCTTC-3’ |
| *L. taiwanensis* (antisense) | 5’-GGTAGACCGCGCATTTTCAGAAACC-3’ |
| *L. reuteri* (sense) | 5’-ACCGAGAACACCGCGTTATTT-3’ |
| *L. reuteri* (antisense) | 5’-CATAACTTAACCTAAACAATCAAAGATTGTCT-3’ |
| All Lactobacillus (sense) | 5’-AGCAGTAGGGAATCTTCCA-3’ |
| All Lactobacillus (antisense) | 5’-CACCGCTACACATGGAG-3’ |
|  |  |
